# Scalp bacterial shift in Alopecia areata

**DOI:** 10.1371/journal.pone.0215206

**Published:** 2019-04-11

**Authors:** Daniela Pinto, Elisabetta Sorbellini, Barbara Marzani, Mariangela Rucco, Giammaria Giuliani, Fabio Rinaldi

**Affiliations:** 1 Giuliani SpA, Milan, Italy; 2 Human Advanced Microbiome Project-HMAP, Milan, Italy; 3 International Hair Research Foundation (IHRF), Milan, Italy; University of Illinois at Urbana-Champaign, UNITED STATES

## Abstract

The role of microbial dysbiosis in scalp disease has been recently hypothesized. However, little information is available with regards to the association between microbial population on the scalp and hair diseases related to hair growth. Here we investigated bacterial communities in healthy and Alopecia areata (AA) subjects. The analysis of bacterial distribution at the genus level highlighted an increase of *Propionibacterium* in AA subjects alongside a general decrease of *Staphylococcus*. Analysis of log Relative abundance of main bacterial species inhabiting the scalp showed a significant increase of *Propionibacterium acnes* in AA subjects compared to control ones. AA scalp condition is also associated with a significant decrease of *Staphylococcus epidermidis* relative abundance. No significant changes were found for *Staphylococcus aureus*. Therefore, data from sequencing profiling of the bacterial population strongly support a different microbial composition of the different area surrounded hair follicle from the epidermis to hypodermis, highlighting differences between normal and AA affected the scalp. Our results highlight, for the first time, the presence of a microbial shift on the scalp of patients suffering from AA and gives the basis for a larger and more complete study of microbial population involvement in hair disorders.

## Introduction

Alopecia areata (AA) is the second most common type of hair loss disorder for human beings. It occurs in the form of a non-scarring alopecia which affects the scalp and, eventually, the entire body [1]. An incidence higher than 2% has been reported for AA, with a lifetime risk of 1.7% both in men and women [2].

For subjects affected by AA, the catagen phase is either extremely short or doesn’t occur at all, and in turn proceeds rapidly to telogen phase. From a clinical point of view, this led to single or several annular or patchy bald lesions usually on the scalp [3,4]. These lesions can extend to the entire scalp (Alopecia totalis) or to the entire pilar area of the body (Alopecia universalis).

The management of AA still remains a challenge and is mainly aimed at containing it. Among treatments currently available [5], in 2012, the British Association of Dermatologists recommended two main treatments with a C grade of recommendation: i) topical and intralesional corticosteroid (limited patchy hair loss); ii) immunotherapy (extensive patchy hair loss and Alopecia totalis/universalis) [6].

Causes behind AA are not yet fully understood, and there have been debates dating back to the beginning of the 1800s. Many associations have been proposed by researchers over the years [7]. However, clinical evidence and association with other immune disorders [8] underline the role of immunity and inflammation in the early development of AA [9–11]. Interestingly, authors [11] reported the efficacy of PRP (Platelet-rich plasma) on AA as a potent anti-inflammatory agent by suppressing cytokine release and limiting local tissue inflammation [11].

Other comimon (common?) recognized offenders are hormonal imbalance, psychological stress, genetic tendencies, other local skin disorders and also nutritional deficiencies [5]. More recently, some authors reported evidence of the link between the gut microbiome and AA [12,13] but little information is currently available as regards microbial communities on the scalp [14,15]. Due to its unique features, the scalp is expected to harbor a specific microbiome, which is expected to play a peculiar role in scalp conditions related to hair growth [16].

In this work, we present data on bacterial communities in healthy and AA subjects, on a sample of Italian population. Our results highlight, for the first time, the presence of a significative bacterial disequilibrium on the scalp of AA subjects compared to healthy population; this disequilibrium also extends in the subepidermal compartments of the scalp.

## Material and methods

### Subjects recruitment

Fifteen healthy and AA subjects, respectively (20–60 years old; 40% male) were recruited from a private Italian dermatological clinic (Milan, Italy).

All subjects have been enrolled under dermatological control. AA subjects have been previously evaluated about their disease history and by means of clinical examinations. Subjects have been enrolled in control population after clinical examinations and in absence of any history of dermatological or scalp disorders.

All enrolled subjects had to meet the following criteria: i) no antibiotics in the 30 days leading up to the sampling; ii) no probiotics in the last 15 days; iii) the last shampoo was performed 48h before sampling; iv) no pregnancy or lactation; v) suffering from other dermatological diseases; vi) no anti-tumor, immunosuppressant or radiation therapy in the last 3 months; vii) no topical or hormonal therapy on the scalp in the last 3 months.

The study was approved by the Ethical Independent Committee for Clinical, not pharmacological investigation in Genoa (Italy) and in accordance with the ethical standards of the 1964 Declaration of Helsinki. All of the volunteers signed the informed consent.

### Swab sample collection

The scalp surface has been sampled by means of swab procedure according to previously reported methods [17,18] with minor modifications. Sterile cotton swabs were soaked for at least 30s in ST solution (NaCl 0.15 M and 0.1% Tween 20) before sampling. A comb was used to separate hair fibers and collect samples from a total area of 16cm^2^ from a different area of the scalp. After collection, the head of each swab was cut and stored in ST solution. Samples from the same subjects were collected together and stored at 4°C until DNA extraction. Sterile cotton swabs placed in ST solution have been used as negative controls.

### Biopsy samples collection

A total of 4 female subjects (two control and two AA, respectively) were also sampled for the microbial community in the subepidermal compartments of the scalp. A 4-mm punch biopsy specimen was collected from each subject. In AA subjects, the specimen was obtained from a well-developed lesion. The sampled area was disinfected prior to the surgery to avoid contamination from surface bacteria. Epidermis, dermis and hypodermis were aseptically separated and stored in Allprotect medium (Qiagen) according to manufacturer conditions until DNA extraction.

### Bacterial DNA extraction

Bacterial DNA from scalp swabs was extracted by mean of QIAamp UCP Pathogen Mini Kit (Qiagen, Milan, Italy) according to manufacturer protocol, with minor modifications [19]. The DNeasy Tissue kit (Qiagen, Milan, Italy) was used for DNA extraction from biopsy specimens. Extracted DNA was finally suspended in DNAse free water and quantified by the QIAexpert system (Qiagen, Milan, Italy) before qRT-PCR and sequencing.

### High throughput 16S amplicon generation, sequencing and analysis

DNA samples extracted from scalp swabs were amplified for the variable region V3-V4 using the universal prokaryotic primers: 341 F CTGNCAGCMGCCGCGGTAA [20,21] and 806bR GGACTACNVGGGTWTCTAAT [22–24] utilizing a modified dual-indexed adapter-linked single step protocol. Library preparation and Illumina MiSeq V3-V4 sequencing were carried out at StarSEQ GmbH, Mainz, Germany, according to the method of Caporaso et al. [25] and Kozich et al., [26] with minor modifications. Amplicons were generated using a high fidelity polymerase (AccuStart II PCR ToughMix, Quantabio, Beverly, MA). The amplicons were then normalized to equimolar concentrations using SequalPrep Plate Normalization Kit (ThermoFisher Scientific, Monza, Italy) and the final concentration of the library was determined using a fluorometric kit (Qubit, Life technologies, Carlsbard, CA, USA). Libraries were mixed with Illumina-generated PhiX control libraries and denatured using fresh NaOH. Runs were performed using Real-Time Analysis software (RTA) v. 1.16.18 and 1.17.22, MiSeq Control Software (MCS) v. 2.0.5 and 2.1.13, varying amounts of a PhiX genomic library control, and varying cluster densities. Four sequencing runs were performed with RTA v. 1.18.54, MCS v. 2.6, a target of 25% PhiX, and 600–700 k/mm2 cluster densities according to Illumina specifications for sequencing of low diversity libraries. We used 25% PhiX to balance the runs and use 600 bp V3 chemistry for sequencing. Basecalls from Illumina High Throughput Sequencing (HTS) machines were converted to fastQ files using bcl2fastq (Illumina) software, v2.20.0.42 and quality control carried out by mean of, v0.11.5. bcl2fastq (Illumina) software, v2.20.0.422. Quality control of fastq reads was carried out using FastQC v0.11.5. The quality trimming of primers and adaptors was carried out using Cutadapt, v. 1.14 [27] and Sickle v. 1.33 [28] toolkits, respectively.

Paired-end reads were assembled using Pandaseq v. 2.11[29] using a threshold of 0.9 and a minimum overlap region length of 50. Clustering was carried out using closed-reference OTU picking and de novo OUT picking protocol of QIIME v1.9 [25] at ≥97% identity.

Greengenes database v13_8 was used as a reference for bacterial taxonomic assignment [30]. Amplicon reads were also analyzed as regards alpha diversity by mean of Shannon index, using QIIME v1.9.

### Bacteria quantification by qRT-PCR

Relative abundance of bacterial DNA of main bacterial species on the scalp was assessed by means of real-time quantitative PCR (RT qPCR). Microbial PCR assay kit (Qiagen, Milan, Italy) with gene-specific primers and TaqMan MGB probe targeting *Propionibacterium acnes*, *Staphylococcus epidermidis* and *Staphylococcus aureus* 16S rRNA gene, respectively, were used. Genbank accession numbers of *16S rRNA* gene sequences for P. acnes, S. aureus and S. epidermidis were ADJL01000005.1, ACOT01000039.1 and ACJC01000191.1, respectively. Samples were mixed with 12.5μL of Microbial qPCR Mastermix, 1 μL of Microbial DNA qPCR Assay, 5ng of genomic DNA sample and Microbial-DNA-free water up to a final volume of 25 μL.

Nine separate PCR reactions are prepared for each sample, including Positive PCR Control, No Template Control, and Microbial DNA Positive Control, as well as the Microbial DNA qPCR Assay. Pan-bacteria (Genebank accession number HQ640630.1) assays that detect a broad range of bacterial species are included to serve as positive controls for the presence of bacterial DNA. Assays for human GAPDH and HBB1 (Genebank accession numbers NT_009759.16 and NT_009237.18, respectively) have been included to determine proper sample collection and used to assess the presence of human genomic DNA in the sample and, eventually, subtracted from calculation. Thermal cycling conditions used were as follows; 95°C for 10 min, 40 cycles of 95°C for 15 sec, 60°C for 2 min. PCR reactions were performed in duplicate using an MX3000p PCR machine (Stratagene, La Jolla, CA). Amplification-curve plotting and calculation of cycle threshold (Ct) values were performed using MX3000p software (v.3; Stratagene) and data were further processed by Excel. ΔΔCt method [31] was used to calculate bacterial load of each swab sample. Obtained values have been used for calculation of Bacterial Load-Fold Change (AA/Healthy subjects). Data is finally expressed as Log of the relative abundance of each sample versus the control group.

### Statistical analysis

Data is expressed as log Relative abundance (RA) ± SEM for qRT-PCR analysis. Results were checked for normal distribution using D'Agostino & Pearson normality test before further analyses. Statistically significant differences on bacterial community between healthy and AA group were determined using Wilcoxon test (p ≤ 0.05). All the comparisons were performed pairwise for each group. Analyses were performed with GraphPad Prism 7.0 (GraphPad Software, Inc., San Diego, CA). P–values equal to or less than 0.05 were considered significant.

## Results

### Microbiota profiling of the scalp in AA subjects

**Fig 1:**
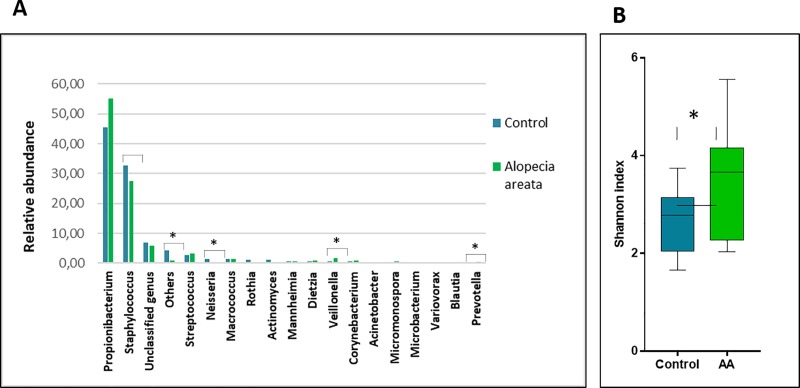
Bacterial profiling in control and AA subjects. **(A)**% of bacteria at genus level in the control and AA groups. Results are presented as the percentage (%) of total sequences, (*p ≤ 0.05). (B) Shannon diversity index for bacterial population observed in control and AA subjects (*p ≤ 0.05).

The human scalp’s bacterial composition of Control (n = 15) and AA (N = 15) subjects have been analyzed by IlluminaSeq (Fig 1). We obtaining about 585,219 and 544,578 high quality reads for the total V3-V4 sequences from control and AA subjects, respectively. About 56.3% of sequences from the control group were assigned to *Actinobacteria* phylum and 35.2% to *Firmicutes*. As regards, AA group *Actinobacteria* were around 57.4% and *Firmicutes* decreased to 29.2%. The analysis of bacterial distribution at the genus level, interestingly, highlighted an increase of *Propionibacterium* from 45.6% to 55.1% in AA subjects. Alongside data showed a general decrease of *Staphylococcus* from 32.6% to 27.4% (Fig 1A). Therefore, the percentage of other less abundant bacteria genus was similar (around 5%) both in control and AA subjects. Alpha-diversity (Shannon diversity index) was significantly higher (p ≤ 0.001) in AA subjects than in the control group (Fig 1B).

### Microbial shift of the scalp surface in AA subjects

As previously reported by other authors [14,15], *P*. *acnes*, *S*. *epidermidis* and *S*. *aureus* are the three major microbial species found on the scalp.

Relative abundance of predominant bacteria on scalps both of control and AA subjects has been analyzed by mean of RT q-PCR. Primers and TaqMan MGB probe specific for 16S region of *P*. *acnes*, *S*. *epidermidis* and *S*. *aureus* were used.

**Fig 2:**
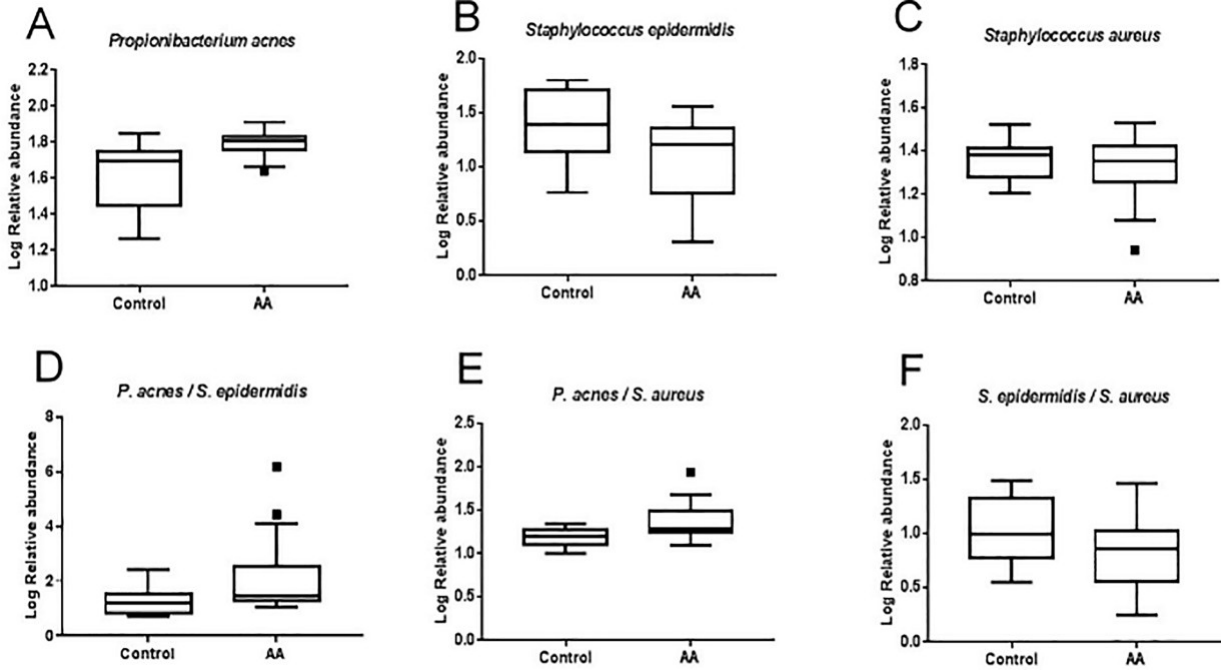
Relative abundance of main bacterial species on the scalp of AA and control subjects by RT qPCR. Box and Whisker comparing the log relative abundance of *P*. *acnes*, *S*. *epidermidis* and *S*. *aureus* collected by swabbing the scalp. (A) Log Relative abundance of *P*. *acnes* in Control and AA subjects. (B) Log Relative abundance of *S*. *epidermidis* in Control and AA subjects. (C) Log Relative abundance of S. aureus in Control and AA subjects. Ratios P. acnes/ S. epidermidis (D), P. acnes/ S. aureus (E) and S. epidermidis / S. aureus (F) in Control and AA subjects. Values are presented as mean +/- SEM, in duplicate. Box-and-Whiskers plot showing median with 25th to 75th percentile. The center line of each box represents the median; data falling outside the whiskers range are plotted as outliers of the data.

Pan bacteria specific targets designed to detect the broadest possible collection of bacteria involved in human biology were used as control. Student's test analysis of log Relative abundance comparing control and AA subjects showed a significant (*p*<0.01) increase of *P*. *acnes* (from 1.6 to 1.8 log RA) in AA subjects compared to control ones (Fig 2A). AA scalp condition is also associated with a significant (*p*<0.05) decrease of *S*. *epidermidis* relative abundance (from 1.4 to 1.01 log RA) (Fig 2B) while no significant changes were found for *S*. *aureus* (Fig 2C).

Microbial shift due to AA is also clear as regards the proportion of bacterial populations analyzed. The ratio *P*. *acnes/ S*. *epidermidis* is significantly higher (*p*<0.05) in AA subjects (mean ratio = 2.1±0.3) compared to control subjects (mean ratio = 1.3±0.1) (Fig 2D). Additionally, the *P*. *acnes/ S*. *aureus* ratio was also significantly higher (*p*<0.01) in AA subjects (mean ratio = 1.4±0.1 vs mean ratio = 1.2±0.1) (Fig 2E). No significative differences were found in the ratio *S*. *epidermidis / S*. *aureus* (Fig 2F).

### AA alteration of bacterial distribution in the subepidermal compartments of the scalp

Two bioptic samples were collected respectively from control and AA subjects and divided in the main subepidermal compartments. Extracted genomic DNAs were analyzed by IlluminaSeq and analyzed for bacterial distribution.

**Fig 3:**
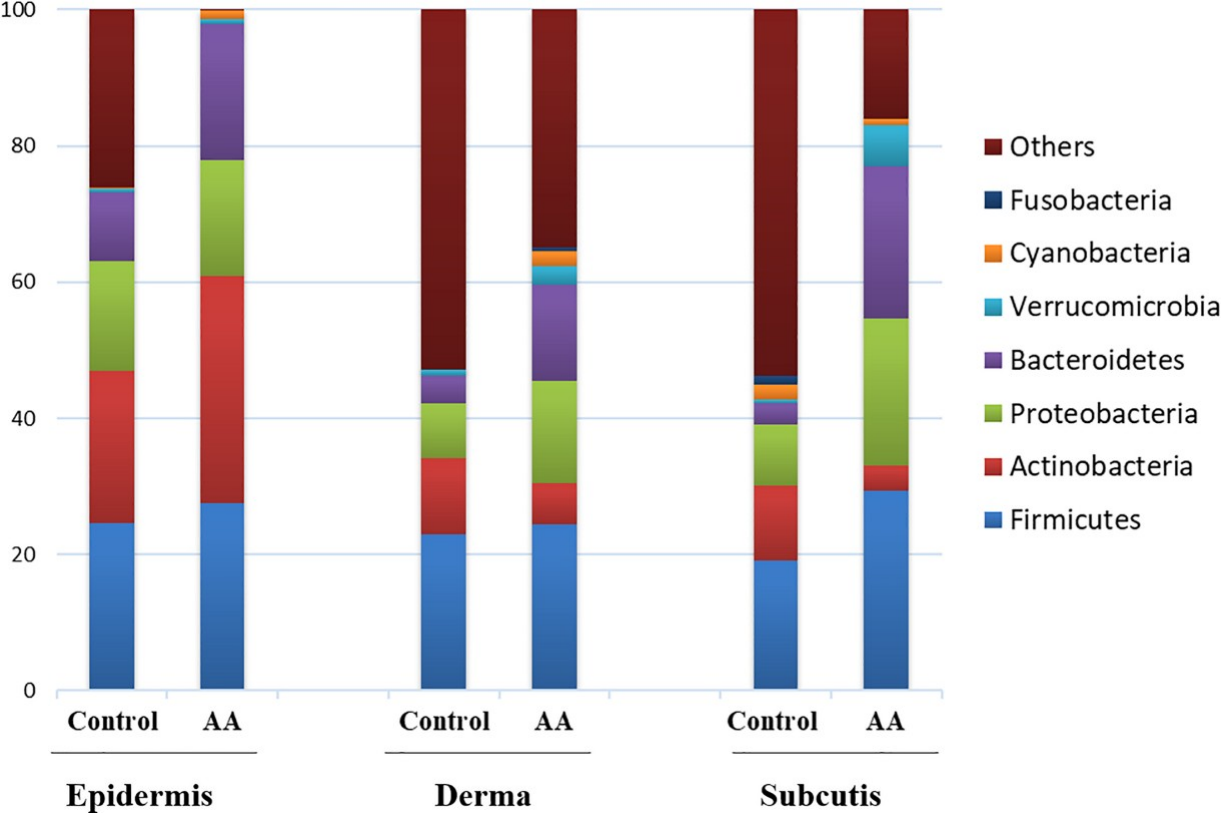
Bacterial profiling of scalp biopsy samples from control and AA subjects. % of bacteria at phylum level in the control and AA groups in the epidermis, dermis and hypodermis. Results are presented as the percentage (%) of total sequences.

Similar proportions of *Firmicutes* (24.6% vs 27.6%) and *Proteobacteria* (16.2% vs 16.9%) were reported in epidermis of both control and AA subjects (Fig 3) while a higher proportion of *Actinobacteria* (33.3% vs 22.4%) and *Bacteroidetes* (20.1% vs 9.9%) were found in AA subjects compared to control (Fig 3). Bacterial community in dermis shifted to a lower proportion of Actinobacteria (6.1% vs 11.3%) in AA subjects while *Proteobacteria* (14.9% vs 8.1%) and *Bacteroidetes* (14.2% vs 4.0%) increased compared to control (Fig 3). Also hypodermis showed a peculiar bacterial distribution which results, also in this case affected by scalp condition. AA subjects showed a significative higher proportion of *Proteobacteria*, *Bacteroidetes* and especially *Firmicutes* than control subjects (Fig 3). In general less variability was observed for bacterial communities in AA subjects and this may reflect in a compromised healthiness of the scalp.

Most interesting, the analysis at species level of bioptic samples highlighted the presence of *Prevotella copri* in both AA samples, in all analyzed compartments.

*Akkermansia muciniphila* was also found (less than 1.5% of total population) in AA subcompartments of the scalp, in particular in the hypodermis.

## Discussion

In this study, we reported, for the first time, the relationship between microbial shift on the scalp and hair growth disorder, in particular, Alopecia areata. We conducted analysis by mean of qRT-PCR and 16S sequencing.

A diversified and abundant microbial community host the skin [32] and this symbiotic relationship results, most of the time, as beneficial for both the host and microbial community [33–35]. Bacteria mainly belong to Corynebacteriaceae, Propionibacteriae, and Staphylococcaceae [36–39] and are differently distributed according to the physiochemical properties of each skin site they host [39,40]. Many scientific published evidence reported the strict correlation between microbial disequilibrium and skin conditions [41–45]. Little is still reported with regards to the microbiome inhabiting the scalp and hair growth disorders [14,15,46]. Clavaud and collaborators [15] and, more recently, Xu et al. [14] reported, the implication of microorganisms in the development of dandruff. Characterization of scalp bacterial species involved in hair disorders such as Alopecia androgenetica, Alopecia areata, and Lichen Planopilaris has been poorly investigated and, only recently, the piece bit of evidence has been reported [16].

We focused our attention on bacterial population of the scalp of healthy and AA subjects looking at main bacterial species on the scalp [15] (*P*. *acnes*, *S*. *aureus*, and *S*. *epidermidis*) and at their reciprocal balancing. We quantified their relative abundance by means of accurate gene-specific primers and probe targeting 16S region, by RT qPCR. Our results are concurrent with Wang’s work [46] highlighting the reciprocal inhibition exerted by bacteria, each other, on the scalp (*Propionibacterium* vs *Staphylococcus* and vice-versa). AA subjects showed an increase in *P*. *acnes* and a decrease of *Staphylococcus*, especially *S*. *epidermidis*, suggesting the role of *Propionibacterium/Staphylococcu*s balancing in AA. A role of *P*. *acnes* with hair casts and Alopecia has previously been hypothesized by Wang and collaborators [46] even though not deeply investigated. *P*. *acnes* is able to synthesize many enzymes involved in the metabolism of porphyrins that, once activated, may contribute to oxidation and follicular inflammation. Therefore, a speculation about the role of the hypoxic condition of the follicular region may be speculated in AA and this may encourage *P*. *acnes* overgrowth. A role of hypoxia has been reported in the progression of other skin condition such as psoriasis [47] and atopic dermatitis [48]. The presence of *A*. *muciniphila*, a strictly anaerobic bacteria, around the hair follicle in analyzed AA subjects may be suggestive of a hypoxic ecosystem in which this bacteria can find favorable growth conditions.

Data from IlluniaSeq profiling also suggested a higher diversity of bacterial species inhabiting the scalp of AA subjects. These results are in line with previous work [15] on other scalp conditions. On the basis of the present and previous results, a link with a higher susceptibility of an unhealthy scalp to be colonized by microorganisms could be postulated but further analysis are needed to understanding the reason behind this high variety.

Beyond the superficial relationship between the microorganism with skin, microbes can also communicate with cells of the subepidermal compartments [49] and are involved also in deep immunological response [50–54]. As reported by Nakatsuji et al., [49] high interpersonal variability was observed as regards epidermal and subepidermal microbial population. In this study, data from sequencing profiling of the bacterial population strongly support a different microbial composition of different area surrounded hair follicle from the epidermis to hypodermis, highlighting differences between normal and AA affected scalp. We can hypothesize the role of this different microbial composition in AA symptoms and manifestations.

Microbial changing at different subepidermal compartment may be linked to an autoimmune component of the pathology as to skin barrier skin disruption, as previously shown for other skin disorders [55].

Most interesting, the analysis at species level of bioptic samples highlighted the peculiar presence of *P*. *copri* and *A*. *muciniphila* in both AA samples, in all analyzed compartments. These findings are very intriguing. The finding of *Prevotella copri* as one of the most abundant bacteria in subepidermal compartments of AA scalp may be linked to the autoimmune component of this hair condition. For example, *P*. *copri* has been found as relevant in the pathogenesis of rheumatoid arthritis [56], another chronic inflammatory autoimmune disorder that can affect other parts of the body including the skin. Therefore the identification of *A*. *muciniphila* in the subepidermal compartments of the scalp of AA subjects could open to new therapeutic approaches in the management of AA. The link between *A*. *muciniphila* and skin disease has been yet discussed as it has been considered a gut signature of psoriasis [57].

The present work reported data from an initial pilot study. Future studies should be aimed at better investigate both the role of microbial community shifts and hypoxia in hair scalp diseases. Also the study of additional factors such as inclusion of samples from non-lesional sites in AA and non-AA subjects and from other baldness disease besides AA and the role should be considered.

## Conclusions

Our study highlighted, for the first time, the presence of a microbial shift on the scalp of patients suffering from AA and gives the basis for a larger and more complete study of microbial population involvement in hair disorders. Therefore, the reported findings as the availability of sophisticated and quick methods to evaluate the microbial composition of the scalp open to new therapeutic approaches in the management of hair disorders.

Larger studies are still needed for a more precise identification of bacterial community on the scalp as for the analysis of fungal component in AA subjects but the results of the present work permit to asses, for the first time, the involvement of microbial changing in hair disorder, in particular AA, also in the subepidermal compartments of the scalp.

## References

[1] Odom RB, Davidsohn IJ, William D, Henry JB, Berger TG. Clinical diagnosis by laboratory methods In: Elston, Dirk M. (Ed.), Andrews’ Diseases of the Skin: Clinical Dermatology. Saunders Elsevier 2006.

[2] Dawber R. Alopecia areata. Monogr Dermatol. 1989; 2:89–102.

[3] Tan E, Tay YK, Goh CL, Chin Giam Y. The pattern and profile of alopecia areata in Singapore—a study of 219 Asians. Int J Dermatol. 2002 11; 41(11):748–53. 12452996 10.1046/j.1365-4362.2002.01357.x

[4] Camacho F. Alopecia areata. Clinical characteristics and dermatopathology In: Trichology: Diseases of the Pilosebaceous Follicle. Aula Medical Group S. A, Madrid; 1997 pp. 440–471.

[5] Syed SA, Sandeep S. Alopecia areata: A review. Journal of the Saudi Society of Dermatology & Dermatologic Surgery. 2013 7; 17(2):37–45.

[6] Messenger AG, McKillop J, Farrant P, McDonagh AJ, Sladden M. British Association of Dermatologists' guidelines for the management of alopecia areata 2012. Br J Dermatol. 2012 5;166(5):916–26. 10.1111/j.1365-2133.2012.10955.x 22524397

[7] McElwee KJ, Gilhar A, Tobin DJ, Ramot Y, Sundberg JP, Nakamura M, et al What causes alopecia areata? Exp Dermaol. 2013;22(9):609–626. 10.1111/exd.12209 PMC4094373 23947678

[8] McDonagh AJ, Tazi-Ahnini R.Epidemiology and genetics of alopecia areata. Clin Exp Dermatol. 2002;27, 405–409. 12190641 10.1046/j.1365-2230.2002.01077.x

[9] Hordinsky M, Ericson M. Autoimmunity: alopecia areata. J Investig Dermatol Symp Proc. 2004 1;9(1):73–8. Review 10.1111/j.1087-0024.2004.00835.x 14870990

[10] Brenner W, Diem E, Gschnait F. Coincidence of vitiligo, alopecia areata, onychodystrophy, localized scleroderma and lichen planus. Dermatologica. 1979;159(4):356–60. 478074 10.1159/000250627

[11] Trink A, Sorbellini E, Bezzola P, Rodella L, Rezzani R, Ramot Y, et al A randomized, double-blind, placebo- and active-controlled, half-head study to evaluate the effects of platelet-rich plasma on alopecia areata. Br J Dermatol.2013 9;169(3):690–4. 10.1111/bjd.12397 23607773

[12] Borde A, Åstrand A. Alopecia areata and the gut-the link opens up for novel therapeutic interventions. Expert Opin Ther Targets. 2018 6;22(6):503–511. 10.1080/14728222.2018.1481504 29808708

[13] Rebello D, Wang E, Yen E, Lio PA, Kelly CR. Hair Growth in Two Alopecia Patients after Fecal Microbiota Transplant. ACG Case Rep J. 2017 9 13;4:e107 10.14309/crj.2017.107 28932754 PMC5599691

[14] Xu Z, Wang Z, Yuan C, Liu X, Yang F, Wang T et al Dandruff is associated with the conjoined interactions between host and microorganisms. Scientific Reports. 2016;6:24877 10.1038/srep24877 27172459 PMC4864613

[15] Clavaud C, Jourdain R, Bar-Hen A, Tichit M, Bouchier C, Pouradier F, et al Dandruff is associated with disequilibrium in the proportion of the major bacterial and fungal populations colonizing the scalp. PLoS One. 8(3):e58203 Erratum in: PLoS One 2013;8(10). 10.1371/journal.pone.0058203 23483996 PMC3590157

[16] Rinaldi F, Pinto D, Marzani B, Rucco M, Giuliani G, Sorbellini E. Human microbiome: What's new in scalp diseases. J Transl Sci. 2018 4; Volume 4(6): 1–4.29657854

[17] Grice EA, Kong HH, Conlan S, Deming CB, Davis J, Young AC, et al Topographical and Temporal Diversity of the Human Skin Microbiome. Science (New York, NY). 2009; 324(5931):1190–1192. 10.1126/science.1171700 19478181 PMC2805064

[18] Paulino LC, Tseng CH, Strober BE, Blaser MJ. Molecular analysis of fungal microbiota in samples from healthy human skin and psoriatic lesions. J Clin Microbiol. 2006 8; 44(8):2933–41. 10.1128/JCM.00785-06 16891514 PMC1594634

[19] Gao Z, Perez-Perez GI, Chen Y, Blaser MJ. Quantitation of Major Human Cutaneous Bacterial and Fungal Populations. Journal of Clinical Microbiology. 2010; 48(10):3575–3581. 10.1128/JCM.00597-10 20702672 PMC2953113

[20] Klindworth A, Pruesse E, Schweer T, Peplies J, Quast C, Horn M, et al Evaluation of general 16S ribosomal RNA gene PCR primers for classical and next-generation sequencing-based diversity studies. Nucleic Acids Res. 2013 1; 7;41(1):e1 10.1093/nar/gks808 22933715 PMC3592464

[21] Takahashi S, Tomita J, Nishioka K, Hisada T, Nishijima M. Development of a Prokaryotic Universal Primer for Simultaneous Analysis of Bacteria and Archaea Using Next-Generation Sequencing. Bourtzis K, ed. PLoS ONE. 2014; 9(8):e105592 10.1371/journal.pone.0105592 25144201 PMC4140814

[22] Apprill A, McNally S, Parsons R, Weber L. Minor revision to V4 region SSU rRNA 806R gene primer greatly increases detection of SAR11 bacterioplankton. Aquat Microb Ecol. 2015;75:129–137.

[23] Parada AE, Needham DM, Fuhrman JA. Every base matters: assessing small subunit rRNA primers for marine microbiomes with mock communities, time series and global field samples. Environ Microbiol. 2016 5;18(5):1403–14. 10.1111/1462-2920.13023 26271760

[24] Walters W, Hyde ER, Berg-Lyons D, Ackermann G, Humphrey G, Parada A. Improved Bacterial 16S rRNA Gene (V4 and V4-5) and Fungal Internal Transcribed Spacer Marker Gene Primers for Microbial Community Surveys. 2016 1(1), e00009–15. 10.1128/mSystems.00009-15 PMC5069754 27822518

[25] Caporaso JG, Lauber CL, Walters WA, Berg-Lyons D, Lozupone CA, Turnbaugh PJ, et al Global patterns of 16S rRNA diversity at a depth of millions of sequences per sample. Proc Natl Acad Sci U S A. 2011; 108(Suppl 1):4516–22. 20534432 10.1073/pnas.1000080107 PMC3063599

[26] Kozich JJ, Westcott SL, Baxter NT, Highlander SK, Schloss PD. Development of a dual-index sequencing strategy and curation pipeline for analyzing amplicon sequence data on the MiSeq Illumina sequencing platform. Appl Environ Microbiol. 2013 9; 79(17):5112–5120. 10.1128/AEM.01043-13 23793624 PMC3753973

[27] Martin M. Cutadapt removes adapter sequences from high-throughput sequencing reads. EMBnet. J. 2011; 17:10–12.

[28] Joshi NA, Fass JN. Sickle: A sliding-window, adaptive, quality-based trimming tool for FastQ files (Version 1.33) [Software]. 2011.

[29] Masella AP, Bartram AK, Truszkowski JM, Brown DG, Neufeld JD. PANDAseq: paired-end assembler for illumina sequences. BMC Bioinformatics. 2012 2;14:13–31. 10.1186/1471-2105-13-31 PMC3471323 22333067

[30] Desantis TZ, Hugenholtz P, Larsen N, Rojas M, Brodie EL, Keller K, et al Greengenes, a chimera-checked 16S rRNA gene database and workbench compatible with ARB. Appl. Environ. Microbiol. 2006; 72: 5069–5072. 10.1128/AEM.03006-05 16820507 PMC1489311

[31] Vigetti D, Viola M, Karousou E, Rizzi M, Moretto P, Genasetti A, et al Hyaluronan-CD44-ERK1/2 regulate human aortic smooth muscle cell motility during aging. J Biol Chem 2008; 283:4448–58. 10.1074/jbc.M709051200 18077444

[32] Findley K, Grice EA. The Skin Microbiome: A Focus on Pathogens and Their Association with Skin Disease. Miller V, ed. PLoS Pathogens. 2014; 10(11):e1004436. 25393405 10.1371/journal.ppat.1004436 PMC4231143

[33] Noble WC. Staphylococci on the skin In The Skin Microflora and Microbial Skin Disease; Noble, W.C., Ed.; Cambridge University Press: London, UK, 2004; pp. 135–152.

[34] Katsuyama M, Ichikawa H, Ogawa S, Ikezawa Z. A novel method to control the balance of skin microflora. Part 1. Attack on biofilm of Staphylococcus aureus without antibiotics. J Dermatol Sci. 2005 6; 38(3):197–205. Epub 2005 Mar 2. Erratum in: J Dermatol Sci. 2005 Sep; 39(3):196. Masako, Katsuyama [corrected to Katsuyama, Masako]; Hideyuki, Ichikawa [corrected to Ichikawa, Hideyuki]; Shigeyuki, Ogawa [corrected to Ogawa, Shigeyuki]; Zenro, Ikezawa [corrected to Ikezawa, Zenro]. 15927813. 10.1016/j.jdermsci.2005.01.006 15927813

[35] Lambers H, Piessens S, Bloem A, Pronk H, Finkel P. Natural skin surface pH is on average below 5, which is beneficial for its resident flora. Int J Cosmet Sci. 2006 10; 28(5):359–70. 10.1111/j.1467-2494.2006.00344.x 18489300

[36] Dethlefsen L, McFall-Ngai M, Relman DA. An ecological and evolutionaryb perspective on human-microbe mutualism and disease. Nature. 2007 10 18; 449(7164):811–8. Review. 10.1038/nature06245 17943117 PMC9464033

[37] Grice EA, Kong HH, Renaud G, Young AC; NISC Comparative Sequencing Program, Bouffard GG, Blakesley RW, Wolfsberg TG, Turner ML, Segre JA. A diversity profile of the human skin microbiota. Genome Res. 2008 7; 18(7):1043–50. 10.1101/gr.075549.107 18502944 PMC2493393

[38] Reid G, Younes JA, Van der Mei HC, Gloor GB, Knight R, Busscher HJ. Microbiota restoration: natural and supplemented recovery of human microbial communities. Nat Rev Microbiol. 2011 1; 9(1):27–38. 10.1038/nrmicro2473 21113182

[39] Human Microbiome Project Consortium. Structure, function and diversity of the healthy human microbiome. Nature. 2012 6 13; 486(7402):207–14. 10.1038/nature11234 22699609 PMC3564958

[40] Kong HH. Skin microbiome: genomics-based insights into the diversity and role of skin microbes. Trends Mol Med. 2011 6; 17(6):320–8. 10.1016/j.molmed.2011.01.013 21376666 PMC3115422

[41] Cogen AL, Nizet V, Gallo RL (2009). Skin microbiota: A source of disease or defence? Br J Dermatol 158, 442–455. 10.1111/j.1365-2133.2008.08437.x PMC2746716 18275522

[42] Brogden NK, Mehalick L, Fischer CL, Wertz PW, Brogden KA. The emerging role of peptides and lipids as antimicrobial epidermal barriers and modulators of local inflammation. Skin Pharmacol Physiol. 2012; 25(4):167–81. 10.1159/000337927 22538862 PMC3564229

[43] Zeeuwen PL, Kleerebezem M, Timmerman HM, Schalkwijk J. Microbiome and skin diseases. Curr Opin Allergy Clin Immunol. 2013 10; 13(5):514–20. 10.1097/ACI.0b013e328364ebeb 23974680

[44] Belkaid Y, Hand TW. Role of the microbiota in immunity and inflammation. Cell. 2014 3 27;157(1):121–41. 10.1016/j.cell.2014.03.011 Review. 24679531 PMC4056765

[45] Wang L, Clavaud C, Bar-Hen A, Cui M, Gao J, Liu Y, et al Characterization of the major bacterial-fungal populations colonizing dandruff scalps in Shanghai, China, shows microbial disequilibrium. Exp Dermatol. 2015 5; 24(5):398–400. 10.1111/exd.12684 25739873

[46] Wang E, Lee JS-S, Hee TH. Is Propionibacterium Acnes Associated with Hair Casts and Alopecia? International Journal of Trichology. 2012;4(2):93–97. 10.4103/0974-7753.96907 23180917 PMC3500081

[47] Rosenberger C, Solovan C, Rosenberger AD, Jinping L, Treudler R, Frei U, et al Upregulation of hypoxia-inducible factors in normal and psoriatic skin. J Invest Dermatol. 2007 10; 127(10):2445–52. 10.1038/sj.jid.5700874 17495954

[48] Manresa MC, Taylor CT. Hypoxia Inducible Factor (HIF) Hydroxylases as Regulators of Intestinal Epithelial Barrier Function. Cell Mo Gastroenterol Hepatol. 2017 2 20; 3(3):303–315. 10.1016/j.jcmgh.2017.02.004 PMC5404106 28462372

[49] Nakatsuji T, Chiang HI, Jiang SB, Nagarajan H, Zengler K, Gallo RL. The microbiome extends to subepidermal compartments of normal skin. Nat Commun. 2013;4:1431 10.1038/ncomms2441 23385576 PMC3655727

[50] Yuki T, Yoshida H, Akazawa Y, Komiya A, Sugiyama Y, Inoue S. Activation of TLR2 enhances tight junction barrier in epidermal keratinocytes. J Immunol. 2011 9 15; 187(6):3230–7. 7. 10.4049/jimmunol.1100058 21841130

[51] Lai Y, Di Nardo A, Nakatsuji T, Leichtle A, Yang Y, Cogen AL, et al Commensal bacteria regulate TLR3-dependent inflammation following skin injury. Nature medicine. 2009; 15(12):1377–1382. 10.1038/nm.2062 19966777 PMC2880863

[52] Lai Y, Cogen AL, Radek KA, Park HJ, Macleod DT, Leichtle A, et al Activation of TLR2 by a small molecule produced by Staphylococcus epidermidis increases antimicrobial defense against bacterial skin infections. J Invest Dermatol. 2010 9; 130(9):2211–21. 10.1038/jid.2010.123 20463690 PMC2922455

[53] Wanke I, Steffen H, Christ C, Krismer B, Götz F, Peschel A, et al Skin commensals amplify the innate immune response to pathogens by activation of distinct signaling pathways. J Invest Dermatol. 2011 2; 131(2):382–90. 10. 10.1038/jid.2010.328 21048787

[54] Naik S, Bouladoux N, Wilhelm C, Molloy MJ, Salcedo R, Kastenmuller W, et al Compartmentalized Control of Skin Immunity by Resident Commensals. Science. 2012 8; 337(6098):1115–9. 10.1126/science.1225152 22837383 PMC3513834

[55] De Benedetto A, Kubo A, Beck LA. Skin barrier disruption: a requirement for allergen sensitization? J Invest Dermatol. 2012; 132:949–963. 10.1038/jid.2011.435 22217737 PMC3279586

[56] Pianta A, Arvikar S, Strle K, Drouin EE, Wang Q, Costello CE, et al Evidence of the Immune Relevance of Prevotella copri, a Gut Microbe, in Patients With Rheumatoid Arthritis. Arthritis Rheumatol. 2017 5; 69(5):964–975. 10.1002/art.40003 27863183 PMC5406252

[57] Tan L, Zhao S, Zhu W, Wu L, Li J, Shen M, Lei L, et al The Akkermansia muciniphila is a gut microbiota signature in psoriasis. Exp Dermatol. 2018 2; 27(2):144–149. 10.1111/exd.13463 29130553

